# 2-(5,7-Dimeth­oxy-4-oxo-4*H*-chromen-2-yl)phenyl 4-toluene­sulfonate

**DOI:** 10.1107/S1600536808022654

**Published:** 2008-07-23

**Authors:** G. Ramachandran, R. Suresh, G. Chakkaravarthi, Charles Christopher Kanakam, V. Manivannan

**Affiliations:** a Department of Chemistry, Valliammai Engineering College, SRM Nagar, Chennai 603 203, India; b Department of Chemistry, Presidency College, Chennai 600 005, India; c Department of Physics, CPCL Polytechnic College, Chennai 600 068, India; d Department of Physics, Presidency College, Chennai 600 005, India

## Abstract

In the crystal structure of the title compound, C_24_H_20_O_7_S, the chromone system makes a dihedral angle of 37.32 (7)° with the adjacent benzene ring. The chromone ring system and the tolyl ring are almost parallel, with a dihedral angle of 4.56 (9)°. The tolyl ring is twisted at an angle of 41.75 (6)° with respect to the benzene ring. Weak intra- and inter­molecular C—H⋯O inter­actions are observed.

## Related literature

For related literature, see: Chenera *et al.* (1993 ▶); Ellis (1997 ▶); Kang *et al.* (2004 ▶); Kooijman *et al.* (1984 ▶); Marx *et al.* (2007 ▶); Puviarasan *et al.* (1998 ▶).
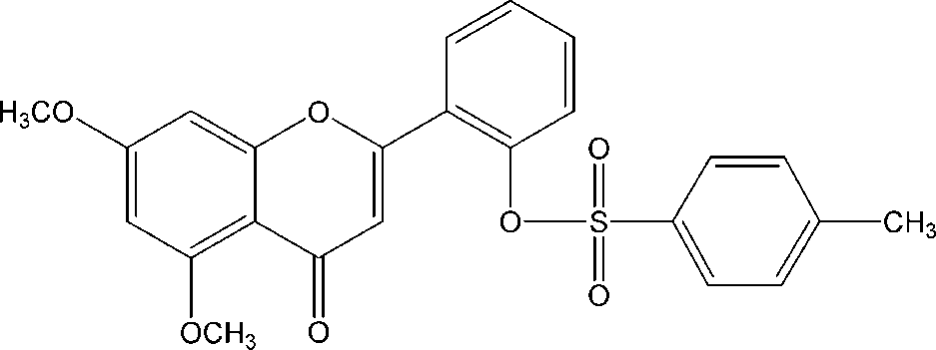


## Experimental

### 

#### Crystal data

C_24_H_20_O_7_S
*M* *_r_* = 452.46
Monoclinic, 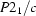

*a* = 7.373 (2) Å
*b* = 21.011 (6) Å
*c* = 13.969 (4) Å
β = 98.972 (5)°
*V* = 2137.6 (10) Å^3^
*Z* = 4
Mo *K*α radiation
μ = 0.20 mm^−1^
*T* = 295 (2) K
0.22 × 0.18 × 0.16 mm

#### Data collection

Bruker Kappa APEXII diffractometer
Absorption correction: multi-scan (**SADABS**; Sheldrick, 1996 ▶) *T*_min_ = 0.958, *T*_max_ = 0.969
24478 measured reflections
5093 independent reflections
3932 reflections with *I* > 2σ(*I*)
*R*_int_ = 0.027

#### Refinement

*R*[*F* ^2^ > 2σ(*F* ^2^)] = 0.050
*wR*(*F* ^2^) = 0.126
*S* = 1.05
5093 reflections
292 parameters
1 restraint
H-atom parameters constrained
Δρ_max_ = 0.27 e Å^−3^
Δρ_min_ = −0.38 e Å^−3^

### 

Data collection: *APEX2* (Bruker, 2004 ▶); cell refinement: *APEX2*; data reduction: *APEX2*; program(s) used to solve structure: *SHELXS97* (Sheldrick, 2008 ▶); program(s) used to refine structure: *SHELXL97* (Sheldrick, 2008 ▶); molecular graphics: *PLATON* (Spek, 2003 ▶); software used to prepare material for publication: *SHELXL97*.

## Supplementary Material

Crystal structure: contains datablocks I, global. DOI: 10.1107/S1600536808022654/is2315sup1.cif

Structure factors: contains datablocks I. DOI: 10.1107/S1600536808022654/is2315Isup2.hkl

Additional supplementary materials:  crystallographic information; 3D view; checkCIF report

## Figures and Tables

**Table 1:** Hydrogen-bond geometry (Å, °)

*D*—H⋯*A*	*D*—H	H⋯*A*	*D*⋯*A*	*D*—H⋯*A*
C6—H6⋯O2	0.93	2.59	2.949 (3)	103
C22—H22⋯O3	0.93	2.56	2.985 (2)	108
C9—H9⋯O2^i^	0.93	2.58	3.240 (2)	129
C12—H12⋯O5^ii^	0.93	2.60	3.510 (2)	168

Symmetry codes: (i) 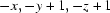; (ii) 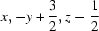.

**Fig. 1.**
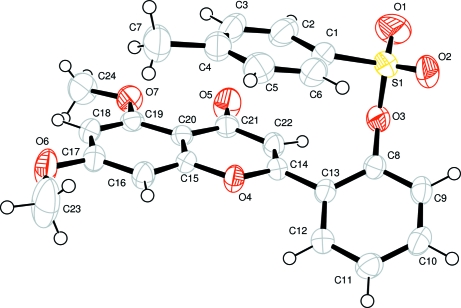
The molecular structure of (I), with atom labels and 50% probability displacement ellipsoids for non-H atoms.

**Fig. 2.**
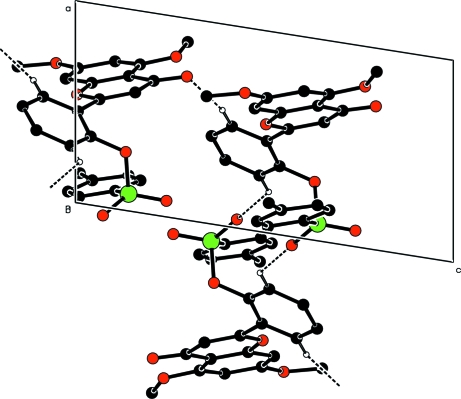
The packing of (I), viewed down the b axis. Hydrogen bonds are shown as dashed lines. H atoms not involved in hydrogen bonding have been omitted.

## supplementary crystallographic information

### Comment

Chromanone derivatives are versatile intermediates for the synthesis of natural products such as brazillin, hematoxylin, ripariochromene, clausenin (Kooijman *et al.*, 1984; Ellis, 1997; Chenera *et al.*, 1993). The title compound, (I), (Fig. 1) has both the sulfonate and flavanone moieties. Hence, it has the structural characteristics of both of them. The geometric parameters of the sulfonate moiety agree with the reported values of similar sulfonates (Kang *et al.*, 2004; Marx *et al.*, 2007; Puviarasan *et al.*, 1998)

The flavanone moiety resembles the chromanone which consists of one benzene ring fused with a six membered oxygen pyranone ring. In chromanone there is an exocyclic double bond at the 4-position of the pyranone ring. In the flavone, there is an endocyclic C═C double bond in the pyranone ring. This brings about a large change in the conformation of pyranone ring.

The pyranone ring is inclined to the benzene C8–C13 ring at an angle of 37.42 (5)°, while the benzene C15—C20 ring and the pyranone ring are co-planar. The flavone and tolyl rings are almost lying in parallel planes, with a dihedral angle of 4.56 (9)°. The tolyl ring makes a dihedral angle of 41.75 (6)° with the benzene C8–C13 ring. The molecular structure is stabilized by the weak intramolecular C—H···O interactions and the crystal packing is stabilized by the weak intermolecular C—H···O interactions (Table 1) (Fig. 2).

### Experimental

Phluroglucinol was converted to phluroaceto phenone by Hoesch reaction. The latter on treatment with *o*-nitro benzoyl chloride, potassium carbonate and acetone afforded nitro flavone. This was methylated and reduced with Tin/con HCl to get amino flavone. Diazotization followed by hydrolysis yielded flavonol. Sulfonylation, in presence of triethyl amine and acetone resulted in 5,7 – dimethoxy-2'-flavonyl-4-sulfonate. Diffraction quality crystals were obtained by recrystallizing the crude product from an ethanol solution.

### Refinement

H atoms were positioned geometrically and refined using riding model with C—H = 0.93 Å and *U*_iso_(H) = 1.2*U*_eq_(C) for aromatic C—H, and C—H = 0.96 Å and *U*_iso_(H) = 1.5*U*_eq_(C) for CH_3_. A rigid bond restraint (*DELU*) was applied for atoms C3 and C4 in the final cycle of the refinement.

### Crystal data

**Table:**

C_24_H_20_O_7_S	*F*_000_ = 944
*M**_r_* = 452.46	*D*_x_ = 1.406 Mg m^−^^3^
Monoclinic, *P*2_1_/*c*	Mo *K*α radiation λ = 0.71073 Å
Hall symbol: -P 2ybc	Cell parameters from 5023 reflections
*a* = 7.373 (2) Å	θ = 1.7–28.0º
*b* = 21.011 (6) Å	µ = 0.20 mm^−^^1^
*c* = 13.969 (4) Å	*T* = 295 (2) K
β = 98.972 (5)º	Block, colourless
*V* = 2137.6 (10) Å^3^	0.22 × 0.18 × 0.16 mm
*Z* = 4	

### Data collection

**Table:**

Bruker Kappa APEXII diffractometer	5093 independent reflections
Radiation source: fine-focus sealed tube	3932 reflections with *I* > 2σ(*I*)
Monochromator: graphite	*R*_int_ = 0.027
*T* = 295(2) K	θ_max_ = 28.0º
ω and φ scans	θ_min_ = 1.8º
Absorption correction: multi-scan(*SADABS*; Sheldrick, 1996)	*h* = −9→9
*T*_min_ = 0.958, *T*_max_ = 0.969	*k* = −27→27
24478 measured reflections	*l* = −17→18

### Refinement

**Table:**

Refinement on *F*^2^	Secondary atom site location: difference Fourier map
Least-squares matrix: full	Hydrogen site location: inferred from neighbouring sites
*R*[*F*^2^ > 2σ(*F*^2^)] = 0.050	H-atom parameters constrained
*wR*(*F*^2^) = 0.126	*w* = 1/[σ^2^(*F*_o_^2^) + (0.0523*P*)^2^ + 0.7841*P*] where *P* = (*F*_o_^2^ + 2*F*_c_^2^)/3
*S* = 1.05	(Δ/σ)_max_ < 0.001
5093 reflections	Δρ_max_ = 0.27 e Å^−^^3^
292 parameters	Δρ_min_ = −0.38 e Å^−^^3^
1 restraint	Extinction correction: none
Primary atom site location: structure-invariant direct methods	

### Fractional atomic coordinates and isotropic or equivalent isotropic displacement parameters (Å^2^)

**Table:**

	*x*	*y*	*z*	*U*_iso_*/*U*_eq_	
S1	0.08488 (8)	0.62413 (2)	0.64071 (4)	0.05905 (18)	
O1	0.0797 (3)	0.61918 (9)	0.74090 (14)	0.0966 (7)	
O2	−0.0424 (2)	0.58983 (7)	0.57276 (15)	0.0778 (5)	
O3	0.28958 (18)	0.59915 (6)	0.63318 (9)	0.0471 (3)	
O4	0.53667 (17)	0.74321 (5)	0.50458 (8)	0.0405 (3)	
O5	0.7115 (2)	0.75407 (7)	0.79509 (10)	0.0666 (4)	
O6	0.6757 (3)	0.95793 (6)	0.44864 (11)	0.0699 (5)	
O7	0.8015 (2)	0.87641 (7)	0.76660 (9)	0.0541 (4)	
C1	0.0892 (3)	0.70374 (9)	0.60523 (15)	0.0476 (5)	
C2	0.1605 (3)	0.74935 (10)	0.67212 (18)	0.0602 (5)	
H2	0.1973	0.7387	0.7368	0.072*	
C3	0.1758 (3)	0.81123 (10)	0.6404 (2)	0.0666 (6)	
H3	0.2228	0.8423	0.6848	0.080*	
C4	0.1234 (3)	0.82809 (10)	0.5447 (2)	0.0651 (6)	
C5	0.0488 (3)	0.78228 (11)	0.48044 (18)	0.0630 (6)	
H5	0.0092	0.7933	0.4161	0.076*	
C6	0.0313 (3)	0.71989 (10)	0.50952 (16)	0.0556 (5)	
H6	−0.0188	0.6892	0.4651	0.067*	
C7	0.1486 (4)	0.89585 (12)	0.5117 (3)	0.0992 (11)	
H7A	0.1668	0.8957	0.4451	0.149*	
H7B	0.2537	0.9144	0.5510	0.149*	
H7C	0.0412	0.9203	0.5180	0.149*	
C8	0.3393 (2)	0.59138 (7)	0.54037 (12)	0.0373 (4)	
C9	0.2781 (2)	0.53817 (8)	0.48695 (14)	0.0430 (4)	
H9	0.2039	0.5085	0.5116	0.052*	
C10	0.3279 (3)	0.52929 (9)	0.39678 (14)	0.0462 (4)	
H10	0.2886	0.4933	0.3606	0.055*	
C11	0.4361 (3)	0.57390 (9)	0.36046 (14)	0.0468 (4)	
H11	0.4669	0.5684	0.2989	0.056*	
C12	0.4992 (3)	0.62672 (8)	0.41444 (13)	0.0427 (4)	
H12	0.5727	0.6563	0.3890	0.051*	
C13	0.4541 (2)	0.63623 (8)	0.50660 (12)	0.0363 (4)	
C14	0.5303 (2)	0.69218 (7)	0.56371 (12)	0.0355 (4)	
C15	0.6059 (2)	0.79985 (7)	0.54323 (12)	0.0355 (4)	
C16	0.6061 (3)	0.84733 (8)	0.47443 (13)	0.0431 (4)	
H16	0.5662	0.8395	0.4090	0.052*	
C17	0.6678 (3)	0.90651 (8)	0.50682 (14)	0.0464 (4)	
C18	0.7306 (3)	0.91772 (9)	0.60496 (14)	0.0463 (4)	
H18	0.7697	0.9583	0.6255	0.056*	
C19	0.7350 (2)	0.86942 (8)	0.67109 (13)	0.0399 (4)	
C20	0.6689 (2)	0.80742 (8)	0.64181 (12)	0.0352 (4)	
C21	0.6642 (3)	0.75290 (8)	0.70711 (13)	0.0416 (4)	
C22	0.5923 (3)	0.69490 (8)	0.65831 (13)	0.0425 (4)	
H22	0.5895	0.6581	0.6949	0.051*	
C23	0.6289 (5)	0.94826 (13)	0.34712 (18)	0.1037 (12)	
H23A	0.7102	0.9173	0.3263	0.156*	
H23B	0.6402	0.9877	0.3139	0.156*	
H23C	0.5047	0.9332	0.3326	0.156*	
C24	0.8834 (4)	0.93587 (12)	0.79739 (17)	0.0718 (7)	
H24A	0.9837	0.9443	0.7628	0.108*	
H24B	0.9282	0.9343	0.8657	0.108*	
H24C	0.7936	0.9691	0.7844	0.108*	

### Atomic displacement parameters (Å^2^)

**Table:**

	*U*^11^	*U*^22^	*U*^33^	*U*^12^	*U*^13^	*U*^23^
S1	0.0666 (3)	0.0429 (3)	0.0766 (4)	−0.0102 (2)	0.0393 (3)	−0.0010 (2)
O1	0.1372 (18)	0.0811 (12)	0.0899 (13)	0.0014 (12)	0.0750 (13)	0.0146 (10)
O2	0.0545 (9)	0.0495 (9)	0.1352 (16)	−0.0205 (7)	0.0327 (9)	−0.0202 (9)
O3	0.0596 (8)	0.0376 (7)	0.0469 (7)	−0.0034 (6)	0.0164 (6)	0.0059 (5)
O4	0.0558 (8)	0.0302 (6)	0.0347 (6)	−0.0089 (5)	0.0051 (5)	−0.0008 (5)
O5	0.1040 (13)	0.0593 (9)	0.0336 (7)	−0.0095 (8)	0.0011 (7)	−0.0001 (6)
O6	0.1180 (14)	0.0340 (7)	0.0570 (9)	−0.0143 (8)	0.0117 (9)	0.0049 (6)
O7	0.0637 (9)	0.0567 (8)	0.0416 (7)	−0.0160 (7)	0.0071 (6)	−0.0158 (6)
C1	0.0453 (10)	0.0370 (9)	0.0641 (13)	−0.0033 (8)	0.0197 (9)	−0.0086 (8)
C2	0.0599 (13)	0.0533 (12)	0.0673 (14)	0.0019 (10)	0.0098 (10)	−0.0162 (10)
C3	0.0526 (12)	0.0453 (12)	0.1012 (14)	−0.0042 (9)	0.0095 (12)	−0.0269 (11)
C4	0.0437 (11)	0.0445 (11)	0.1110 (15)	0.0029 (9)	0.0248 (11)	0.0005 (12)
C5	0.0588 (13)	0.0569 (13)	0.0756 (15)	0.0076 (10)	0.0176 (11)	0.0057 (11)
C6	0.0522 (12)	0.0483 (11)	0.0675 (14)	−0.0034 (9)	0.0130 (10)	−0.0119 (10)
C7	0.0805 (18)	0.0483 (14)	0.179 (3)	0.0009 (13)	0.054 (2)	0.0132 (17)
C8	0.0411 (9)	0.0301 (8)	0.0408 (9)	0.0028 (7)	0.0066 (7)	0.0045 (7)
C9	0.0416 (9)	0.0295 (8)	0.0569 (11)	−0.0021 (7)	0.0046 (8)	0.0035 (7)
C10	0.0445 (10)	0.0349 (9)	0.0569 (11)	−0.0015 (7)	0.0004 (8)	−0.0103 (8)
C11	0.0493 (11)	0.0479 (10)	0.0434 (10)	−0.0011 (8)	0.0081 (8)	−0.0098 (8)
C12	0.0464 (10)	0.0375 (9)	0.0457 (10)	−0.0055 (7)	0.0113 (8)	−0.0018 (7)
C13	0.0381 (9)	0.0298 (8)	0.0407 (9)	−0.0002 (6)	0.0051 (7)	−0.0001 (7)
C14	0.0385 (9)	0.0307 (8)	0.0384 (9)	−0.0018 (6)	0.0099 (7)	0.0018 (6)
C15	0.0384 (9)	0.0303 (8)	0.0388 (9)	−0.0031 (6)	0.0090 (7)	−0.0047 (7)
C16	0.0567 (11)	0.0361 (9)	0.0360 (9)	−0.0053 (8)	0.0055 (8)	−0.0011 (7)
C17	0.0577 (11)	0.0334 (9)	0.0497 (11)	−0.0048 (8)	0.0134 (9)	0.0008 (8)
C18	0.0532 (11)	0.0342 (9)	0.0538 (11)	−0.0094 (8)	0.0152 (9)	−0.0120 (8)
C19	0.0378 (9)	0.0427 (9)	0.0409 (9)	−0.0039 (7)	0.0115 (7)	−0.0106 (7)
C20	0.0348 (8)	0.0369 (8)	0.0352 (8)	−0.0023 (6)	0.0094 (6)	−0.0047 (7)
C21	0.0455 (10)	0.0437 (10)	0.0359 (9)	−0.0012 (8)	0.0073 (7)	−0.0010 (7)
C22	0.0511 (10)	0.0357 (9)	0.0409 (10)	−0.0031 (7)	0.0080 (8)	0.0048 (7)
C23	0.196 (4)	0.0525 (14)	0.0567 (15)	−0.0225 (18)	0.0020 (18)	0.0179 (12)
C24	0.0765 (16)	0.0766 (16)	0.0605 (14)	−0.0301 (13)	0.0054 (12)	−0.0266 (12)

### Geometric parameters (Å, °)

**Table:**

S1—O1	1.4099 (19)	C9—C10	1.379 (3)
S1—O2	1.4226 (18)	C9—H9	0.9300
S1—O3	1.6168 (15)	C10—C11	1.378 (3)
S1—C1	1.746 (2)	C10—H10	0.9300
O3—C8	1.411 (2)	C11—C12	1.381 (3)
O4—C14	1.3588 (19)	C11—H11	0.9300
O4—C15	1.3720 (19)	C12—C13	1.394 (2)
O5—C21	1.224 (2)	C12—H12	0.9300
O6—C17	1.359 (2)	C13—C14	1.481 (2)
O6—C23	1.421 (3)	C14—C22	1.331 (2)
O7—C19	1.356 (2)	C15—C16	1.385 (2)
O7—C24	1.425 (2)	C15—C20	1.392 (2)
C1—C6	1.381 (3)	C16—C17	1.376 (2)
C1—C2	1.384 (3)	C16—H16	0.9300
C2—C3	1.384 (3)	C17—C18	1.397 (3)
C2—H2	0.9300	C18—C19	1.369 (3)
C3—C4	1.378 (4)	C18—H18	0.9300
C3—H3	0.9300	C19—C20	1.428 (2)
C4—C5	1.372 (3)	C20—C21	1.468 (2)
C4—C7	1.517 (3)	C21—C22	1.455 (2)
C5—C6	1.384 (3)	C22—H22	0.9300
C5—H5	0.9300	C23—H23A	0.9600
C6—H6	0.9300	C23—H23B	0.9600
C7—H7A	0.9600	C23—H23C	0.9600
C7—H7B	0.9600	C24—H24A	0.9600
C7—H7C	0.9600	C24—H24B	0.9600
C8—C9	1.380 (2)	C24—H24C	0.9600
C8—C13	1.397 (2)		
			
O1—S1—O2	120.53 (12)	C11—C12—C13	120.71 (16)
O1—S1—O3	102.15 (11)	C11—C12—H12	119.6
O2—S1—O3	108.53 (9)	C13—C12—H12	119.6
O1—S1—C1	110.91 (11)	C12—C13—C8	117.38 (15)
O2—S1—C1	109.59 (11)	C12—C13—C14	119.07 (15)
O3—S1—C1	103.52 (8)	C8—C13—C14	123.54 (15)
C8—O3—S1	118.44 (11)	C22—C14—O4	122.06 (15)
C14—O4—C15	119.42 (13)	C22—C14—C13	127.71 (15)
C17—O6—C23	117.19 (16)	O4—C14—C13	110.19 (14)
C19—O7—C24	117.60 (16)	O4—C15—C16	113.08 (14)
C6—C1—C2	120.7 (2)	O4—C15—C20	122.33 (14)
C6—C1—S1	119.57 (15)	C16—C15—C20	124.59 (15)
C2—C1—S1	119.57 (17)	C17—C16—C15	117.29 (16)
C1—C2—C3	118.4 (2)	C17—C16—H16	121.4
C1—C2—H2	120.8	C15—C16—H16	121.4
C3—C2—H2	120.8	O6—C17—C16	124.38 (17)
C4—C3—C2	121.8 (2)	O6—C17—C18	114.47 (16)
C4—C3—H3	119.1	C16—C17—C18	121.14 (17)
C2—C3—H3	119.1	C19—C18—C17	120.43 (16)
C5—C4—C3	118.5 (2)	C19—C18—H18	119.8
C5—C4—C7	121.0 (3)	C17—C18—H18	119.8
C3—C4—C7	120.5 (3)	O7—C19—C18	123.41 (16)
C4—C5—C6	121.2 (2)	O7—C19—C20	115.82 (16)
C4—C5—H5	119.4	C18—C19—C20	120.77 (16)
C6—C5—H5	119.4	C15—C20—C19	115.71 (15)
C1—C6—C5	119.2 (2)	C15—C20—C21	119.27 (15)
C1—C6—H6	120.4	C19—C20—C21	125.01 (15)
C5—C6—H6	120.4	O5—C21—C22	120.86 (17)
C4—C7—H7A	109.5	O5—C21—C20	125.27 (17)
C4—C7—H7B	109.5	C22—C21—C20	113.86 (15)
H7A—C7—H7B	109.5	C14—C22—C21	123.01 (16)
C4—C7—H7C	109.5	C14—C22—H22	118.5
H7A—C7—H7C	109.5	C21—C22—H22	118.5
H7B—C7—H7C	109.5	O6—C23—H23A	109.5
C9—C8—C13	121.86 (16)	O6—C23—H23B	109.5
C9—C8—O3	118.74 (15)	H23A—C23—H23B	109.5
C13—C8—O3	119.35 (14)	O6—C23—H23C	109.5
C10—C9—C8	119.51 (16)	H23A—C23—H23C	109.5
C10—C9—H9	120.2	H23B—C23—H23C	109.5
C8—C9—H9	120.2	O7—C24—H24A	109.5
C11—C10—C9	119.76 (16)	O7—C24—H24B	109.5
C11—C10—H10	120.1	H24A—C24—H24B	109.5
C9—C10—H10	120.1	O7—C24—H24C	109.5
C10—C11—C12	120.73 (17)	H24A—C24—H24C	109.5
C10—C11—H11	119.6	H24B—C24—H24C	109.5
C12—C11—H11	119.6		
			
O1—S1—O3—C8	173.84 (13)	C12—C13—C14—C22	−140.30 (19)
O2—S1—O3—C8	45.51 (14)	C8—C13—C14—C22	39.1 (3)
C1—S1—O3—C8	−70.87 (13)	C12—C13—C14—O4	37.4 (2)
O1—S1—C1—C6	−157.58 (18)	C8—C13—C14—O4	−143.28 (16)
O2—S1—C1—C6	−22.09 (19)	C14—O4—C15—C16	179.20 (15)
O3—S1—C1—C6	93.54 (17)	C14—O4—C15—C20	−0.6 (2)
O1—S1—C1—C2	26.1 (2)	O4—C15—C16—C17	178.01 (16)
O2—S1—C1—C2	161.64 (16)	C20—C15—C16—C17	−2.2 (3)
O3—S1—C1—C2	−82.74 (17)	C23—O6—C17—C16	−4.5 (3)
C6—C1—C2—C3	−1.3 (3)	C23—O6—C17—C18	174.7 (2)
S1—C1—C2—C3	174.95 (16)	C15—C16—C17—O6	−179.71 (19)
C1—C2—C3—C4	−0.4 (3)	C15—C16—C17—C18	1.1 (3)
C2—C3—C4—C5	2.1 (3)	O6—C17—C18—C19	−178.11 (18)
C2—C3—C4—C7	−177.7 (2)	C16—C17—C18—C19	1.1 (3)
C3—C4—C5—C6	−2.1 (3)	C24—O7—C19—C18	−5.0 (3)
C7—C4—C5—C6	177.7 (2)	C24—O7—C19—C20	174.65 (18)
C2—C1—C6—C5	1.3 (3)	C17—C18—C19—O7	177.12 (17)
S1—C1—C6—C5	−174.92 (16)	C17—C18—C19—C20	−2.5 (3)
C4—C5—C6—C1	0.4 (3)	O4—C15—C20—C19	−179.33 (15)
S1—O3—C8—C9	−77.99 (17)	C16—C15—C20—C19	0.9 (2)
S1—O3—C8—C13	104.39 (16)	O4—C15—C20—C21	0.9 (2)
C13—C8—C9—C10	−1.4 (3)	C16—C15—C20—C21	−178.93 (17)
O3—C8—C9—C10	−178.93 (15)	O7—C19—C20—C15	−178.12 (15)
C8—C9—C10—C11	−0.8 (3)	C18—C19—C20—C15	1.5 (2)
C9—C10—C11—C12	1.7 (3)	O7—C19—C20—C21	1.6 (2)
C10—C11—C12—C13	−0.4 (3)	C18—C19—C20—C21	−178.72 (17)
C11—C12—C13—C8	−1.7 (3)	C15—C20—C21—O5	−178.16 (19)
C11—C12—C13—C14	177.69 (16)	C19—C20—C21—O5	2.1 (3)
C9—C8—C13—C12	2.6 (3)	C15—C20—C21—C22	0.5 (2)
O3—C8—C13—C12	−179.87 (15)	C19—C20—C21—C22	−179.25 (16)
C9—C8—C13—C14	−176.78 (16)	O4—C14—C22—C21	2.8 (3)
O3—C8—C13—C14	0.8 (2)	C13—C14—C22—C21	−179.82 (17)
C15—O4—C14—C22	−1.2 (2)	O5—C21—C22—C14	176.42 (19)
C15—O4—C14—C13	−179.02 (13)	C20—C21—C22—C14	−2.3 (3)

### Hydrogen-bond geometry (Å, °)

**Table:**

*D*—H···*A*	*D*—H	H···*A*	*D*···*A*	*D*—H···*A*
C6—H6···O2	0.93	2.59	2.949 (3)	103
C22—H22···O3	0.93	2.56	2.985 (2)	108
C9—H9···O2^i^	0.93	2.58	3.240 (2)	129
C12—H12···O5^ii^	0.93	2.60	3.510 (2)	168

Symmetry codes: (i) −*x*, −*y*+1, −*z*+1; (ii) *x*, −*y*+3/2, *z*−1/2.
